# A Comparative Analysis of the Physical Modelling of Two Methods of Balls Separation

**DOI:** 10.3390/ma14237126

**Published:** 2021-11-23

**Authors:** Łukasz Wójcik, Zbigniew Pater, Tomasz Bulzak, Janusz Tomczak, Konrad Lis

**Affiliations:** Mechanical Faculty, Lublin University of Technology, 36 Nadbystrzycka Str., 20-618 Lublin, Poland; z.pater@pollub.pl (Z.P.); t.bulzak@pollub.pl (T.B.); j.tomczak@pollub.pl (J.T.); k.lis@pollub.pl (K.L.)

**Keywords:** skew wedge rolling, physical modelling, plasticine

## Abstract

The article presents the results of model tests with which a comparative analysis of two methods of ball separation during the skew rolling process was carried out. A verification of the results obtained in the physical modelling process with the results obtained in the real process of skew ball rolling was also carried out. During the physical modelling, the effect of changing the ball separation method on the quality of the products obtained, variations in maximum torque values and maximum radial forces were analyzed. In the case of real tests, the results were verified with the results of physical modelling, in which the surface quality and torque values for one of the tool sets were compared. Physical modelling was used to verify the differences between the two methods of ball separation. Commercial plasticine based on synthetic wax from the manufacturer PRIMO was used as a model material for physical analysis. The plasticine used for testing was cooled to 0 °C and the cooling process took 24 h. The tools used for the physical modelling were 3D printed and the material used was ABS. The method of physical modelling using plasticine as a model material allows for a correct analysis of hot metal forming processes.

## 1. Introduction

Currently, produced steel balls are used in the engineering, mining and processing industries. In the engineering industry, the balls are mainly used for the production of rolling bearings, while the mining and processing industries use them to produce grinding bowls for ball mills for grinding, for example, coal, metal ores, cement, and so forth.

Balls used for the production of bearings are mainly made with the die-forging method due to the demand for high accuracy and quality balls. On the other hand, balls used for ball mills can be made by forging [1,2], casting [3,4], cross-wedge rolling [5,6,7] and skew (helical) rolling [8,9,10]. Balls used in the mining and processing industries do not need high precision and high accuracy, for example, the industry standard BN-84/0660-03 shows that balls with a diameter of 30 mm to 100 mm made by helical rolling must maintain a dimensional deviation of ±3 mm maximum [11].

**Figure 1:**
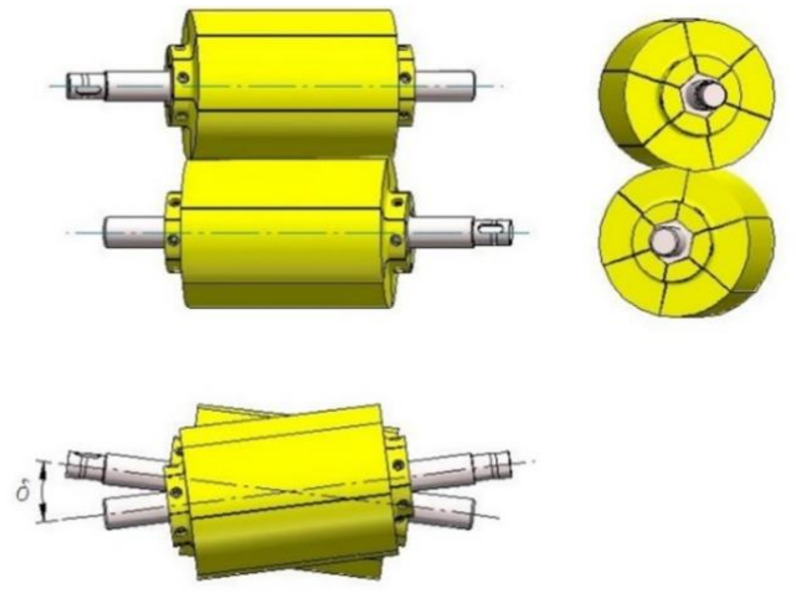
Diagram of the skew rolling process.

The process of helical (skew) ball billet material is based on forming the billet material (bar) into the form of balls between two skewed rollers [12]. A diagram of the roll positioning in the skew ball rolling process is shown in Figure 1.

The workpiece used to produce spheres is a solid bar, while cylindrical tools have a helical shape pass around the circumference of the cylinder with the target shape of the obtained ball. The shaped passes are variable in shape and pitch. When the balls are formed, the rotating tools grasp the input material with their flanges, then start to rotate it around its own axis and shape it according to the shape of the helical pattern. As a result of a change in the width of the tooling flanges, the batch is subjected to axial crushing until it reaches the ball blanks connected by a bridge, which is torn or cut off in the last stage of forming. The tools can be multi-rolled, the use of several rolls of forming pass allows the increase of the number of produced balls at one roll rotation [11,12].

In order to produce balls with the correct shape and a high surface quality in the skew (helical) rolling process, three main conditions must be met in tool design. The first condition specifies that the volume of material enclosed in the pass should be constant during forming. The second condition specifies that the changes in the profile and dimensions of the flange of the blank should be equal to the elongation of the impression to be compressed. In the optimum variant, the length of the compressed bridge should be equal to the width of the top of the flange. If the increase of the flange width is smaller than the elongation of the bridge, the material will move away from the pass and a lapping will form on the surface of the pass, which, in the further process, will be rolled into a teeming arrest. On the other hand, if the variation in flange width is greater than the elongation, axial tensile stresses will be generated in the compressed bridge, which may result in its breaking. The last condition is that the closing of the billet should be achieved over the minimum possible forming distance. Too long a forming path has a negative effect on the cracks within the formed product [5,6,7,8,9].

Physical modelling was used for laboratory testing of ball separation methods in the skew (helical) rolling process. The use of physical modelling allows a more accurate analysis of the ball separation process than numerical methods conducted by finite element calculations. FEM is a very good method for evaluating stresses and deformations that occur within a formed material. FEM can be used to determine process limitations such as underfilling, overfilling and sliding during rolling. However, the determination of the quality of the obtained ball surface and the problem associated with their separation is a difficult issue to analyze, mainly due to limitations in FEM analysis programs. Very often, a simulation is introduced with a simplification in the form of skipping the separation stage [5,13,14].

The idea of physical modelling is to replace the real material with a model material, which allows the hot metal forming process to be modelled [15,16,17,18,19].

The use of physical modelling allows for a reduction in testing time and testing costs by using laboratory machines to replace real materials and machines used in industry. Physical modelling makes it possible to observe the process while it is in progress, for example, by using transparent tool materials and by using materials that can be formed at room temperature. In contrast, the main disadvantages of physical modelling include the time-consuming preparation of samples [16,17,18,19,20].

The physical modelling of metal forming processes is based on five similarity conditions [16,21,22,23,24]. The first similarity condition is the similarity of the flow curves of the model material to the real material, that is, the flow curve of the material should be qualitatively similar to the flow curve of the real material. Based on the first condition, the similarity coefficient *λ* between the model material and the real material is calculated. The similarity coefficient is calculated using the relation described by Equation (1).

(1) $$\lambda =\frac{\int_{0}^{\varepsilon }\sigma_{F\;real}d\varepsilon }{\int_{0}^{\varepsilon }\sigma_{F\;model}d\varepsilon }$$

where *λ*—Similarity factor of the flow curve of the model material to the flow curve of the real material, [-]; *σ_F real_*—flow stress of the real material, [MPa]; *σ_F model_*—flow stress of the model material, [MPa].

The next condition of similarity that must be met is the similarity of friction conditions between the model material and the real material. The values of factors and friction coefficients are recommended to be the same for both pairs of tool materials and batches for physical and real modelling. The third similarity condition is the tool shape similarity condition, that is, the shape of the tools should be the same, while the geometric size of the tool can be scaled by selecting an appropriate scale factor. The next similarity condition used in physical testing processes is the similarity of the kinematics of the model process to the real process, while the last similarity criterion is the thermal similarity of the process.

Physical modelling can be divided into two groups. The first group is static physical modelling, while the second group is dynamic modelling.

The model material used for physical testing should have similar flow characteristics and lower stress values than the real material. This type of material allows the use of model materials for forming tools.

Model materials used for physical modeling can be divided into two groups [14,15,25,26,27,28,29]. The first group includes non-metallic materials, while the second group includes metallic materials. The use of materials from the non-metallic group allows for the application of laboratory machines with much reduced energy requirements for tests. In the group of non-metallic materials, the following materials are included: plasticine, natural resins, synthetic resins, natural waxes, synthetic waxes (filia), mixtures of resins and waxes. The group of metallic materials is mainly based on aluminum alloys, lead, tin and copper [16,17,27,28,29,30,31].

In physical modeling, you can also replace the material from which the tools are made with model materials. Tools made of light metal alloys, wood or plastics are very often used for physical examinations [32,33,34].

In recent times, plastics have been the most commonly used plastics for model tests, with which it is easy to make model tools of the same shape as the real tool. Model materials based on plastics include polycarbonates, PET and resins that allow for the analysis of material flow during the process due to their transparency. Plastics that we can use for physical modelling include: PVC, ABS, PLA, PTFE, we can use these materials to make tools with modern 3D printing methods [32,33,34,35,36].

Commercial Plasticine was used as a model material for the physical modelling presented in this publication. Plasticine is a material characterized by low strength and good plasticity. It is a cheap and commonly available material. Plasticine is a mixture of mainly the synthetic and natural waxes and oils. Additionally, additives are added to the wax mixture in order to remove the effect of self-hardening and the effect of too high viscosity. Natural clays, dyes, kaolin, talc, sulphur and other fillers of mineral origin are used as additives to plasticine [17,37,38,39,40].

Commercial plasticine is a material that can be used for multiple studies and the use of its colored types allows for the construction of multi-layered models. Plasticine as a model material has been used many times in the physical modelling of metal forming processes such as forging [41,42,43,44], rolling [45,46] and extrusion [47,48,49,50].

Physical modelling of rolling processes dates to the 1960s. Gockyu’s research team [51] was one of the first to carry out a physical study of the forge rolling process, where they analyzed the rolling process and how the material flows during shaping.

On the other hand, the laboratory testing of the cross and wedge rolling process using model material was one of the first conducted by the Awano team [52]. Physical modelling of the processes of cross-wedge rolling was conducted in recent years by the team of Wójcik and Pater, where the model material used is commercial plasticine formed at different temperatures [35,36].

The process of skew rolling has been studied by, among others, the scientific team of Shih [53]. They used plasticine to study skew rolling with three and four rolls.

The skew rolling of balls first physically modelled by Chitkar’s team [54], who in their publication presented an analysis of the effect of changing tool angle on the shape and quality of the final product. For their study, they used white and black plasticine formed at room temperature.

After a long period, the subject of skew rolling was taken up by Pater, who analyzed and compared the results of the skew rolling of balls obtained from the real and model processes [35].

As a result of the analysis of laboratory studies of oblique ball rolling processes with screw rollers carried out so far, it has been observed that authors have not focused on the problem of modelling the ball separation process during rolling and it has also been observed that the methods of ball separation significantly affect the quality of products obtained and the lifetime of tools. Therefore, it was considered appropriate in our research to undertake the subject of comparative analysis of two different methods of ball separation using physical modelling with verification in a real process on one of the tested sets of tools.

## 2. Model Materials

A modelling material of the commercial plasticine type, which was produced by the manufacturer PRIMO (Italy, Via Bassa)—who manufacture products based on synthetic waxes—was used for physical modeling tests of the hot skew ball rolling process. White and black plasticine was used for the modelling analysis. The plasticine used is a non-metallic material based on synthetic wax, with clays, oils and color pigments used as additives.

The model material had been previously tested with regard to plastometric tests, the determination of the coefficient and factor of friction, the determination of the Cockroft–Latham fracture criterion and the propagation of cracks as a result of the Mannesmann effect.

Analyses of the model material were carried out in the temperature range from 0 °C to 20 °C. Samples of the model material used for testing were previously prepared according to the author’s procedure. The process of preparing samples for model tests is based on the following stages. The first stage of sample preparation is the initial manual preparation of commercial plasticine supplied by the manufacturer in the form of billets weighing approximately 0.5 kg. This process consists of repeated processing of the plasticine, preheated to approx. 35 °C. The use of manual mixing eliminates any air voids that may have formed during production of the plasticine billets and thus negatively affects the quality of the specimens prepared. The next stage is the preparation of samples of shape (usually extrusion or rolling). The final stage of preparing samples for model tests is a process of 24-h cooling of the prepared materials to the temperature at which the component will be formed. A period of 24 h allows us to obtain the same temperature in the whole volume of the samples.

Plastometric tests of plasticine were carried out using the static compression method [55]. The laboratory test was carried out according to the PN-57/H 04320 standard with the use of an INSTRON 3369 testing machine (Instron, Norwood, MA, USA) with Teflon spacers, for five temperatures: 0 °C, 5 °C, 10 °C, 15 °C and 20 °C. The tests were carried out at three different strain rates. Based on the results obtained, plastometric equations were developed to describe the behavior of commercial white and black plasticine. A constitutive equation was developed that is a function of strain, strain rate and forming temperature. Plastometric Equation (2) presents the equation describing the plasticine, while Table 1 shows the equation constants for the different variants of the plasticine model material,

(2) $$\sigma_{F}=C\varepsilon^{n_{1}}e^{n_{2}\varepsilon }{\dot{\varepsilon }}^{\left(m+bT\right)}e^{aT},$$

where: σ_F_—flow stress [MPa]; ε—strain [-]; $\dot{\varepsilon }$—strain rate [1/s]; C, n_1_, n_2_, m, b, a—constant

**Figure 2:**
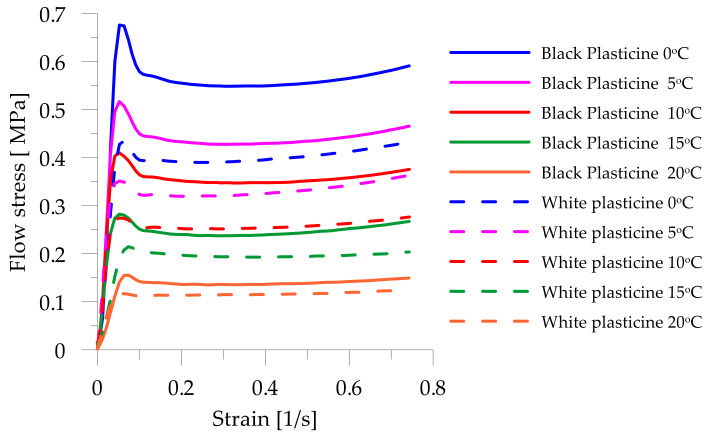
Commercial plasticine flow stress curves.

The flow curves for plasticine are shown and compared in Figure 2. It was observed that a reduction in temperature increases the flow stress of the material and the use of different color pigments affects the differences between the flow curves.

The next test to which the plasticine used for physical testing was subjected was the determination of the size of the cracking criterion according to Cockroft–Latham [56,57]. The laboratory test was carried out using a static tensile test. The test was carried out in two stages, the first stage was to prepare the specimens and conduct the static tensile test using the INSTRON 3369 testing machine. Special jaws were designed for the machine to hold the plasticine specimens; these jaws were made by 3D printing from ABS plastic. In the second stage of the research, a numerical analysis was carried out using DEFORM 3D software. Tensile specimens of the same shape as tensile specimens in the real test were simulated. Data obtained from previous plastometric tests were used as the material model for the simulation. The obtained Cockroft–Latham criterion results were summarized and presented in Table 2. It was observed that the white plasticine obtained lower values than the black plasticine, and an increase in temperature also caused an increase in the fracture criterion values. A comparison with hot-formed C45 steel is also presented, where the similarity of results to the values obtained for formed steel was observed.

Another study to characterize the properties of PRIMO plasticine as a model material was the determination of the coefficient of friction between the plasticine and tools made of plastic [58]. The tools for the study were made of ABS by 3D printing. Laboratory tests were carried out using a CGD-E2000 chain-drawing machine, which was equipped with an AXIS measuring system. Tests were carried out on materials that were formed in the temperature range from 0 °C to 20 °C, using the Coulomb friction model. Physical modelling was carried out for dry friction conditions and with Teflon oil. Static and dynamic friction coefficients were determined based on force diagrams obtained during the tests. The coefficients of friction obtained are presented in Table 3.

Based on the obtained values of friction coefficients, it was found that, for tests performed under conditions of dry friction, the friction coefficients are outside the range, while the application of Teflon oil allowed a significant reduction of the friction coefficient. High values of friction coefficients are caused by a specific property of the model material, that is, high material viscosity. The roughness of the tools obtained from 3D printing also had a large effect on the friction coefficient.

**Figure 3:**
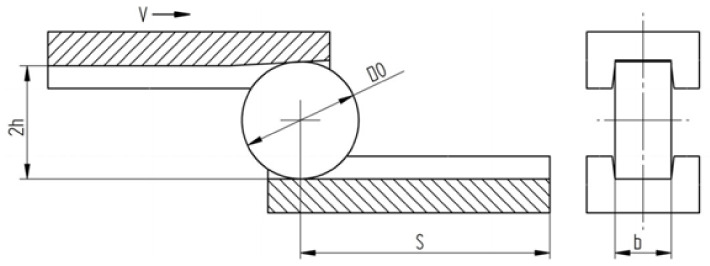
Scheme of the process of compression in a channel [58].

Another study was a model analysis of the phenomenon of cracking as a result of the Mannesmann effect [58]. White and black plasticine was also used in the study, shaped on channel-shaped tools, while the material was formed in the temperature range from 0 °C to 20 °C. The tools used in the study were made of ABS by 3D printing. The forming procedure consisted in putting a disc of material of diameter D0 into the channel of the lower tool at a suitable distance from its end S, then the upper tool moving with speed v formed the disc into a dimension equal to 2 h. A diagram of the forming process is shown in Figure 3.

Based on the test results, a comparative analysis of the model material was carried out with C45, 50HS and R260 steels hot-formed in the temperature range 950 °C–1050 °C.

**Figure 4:**
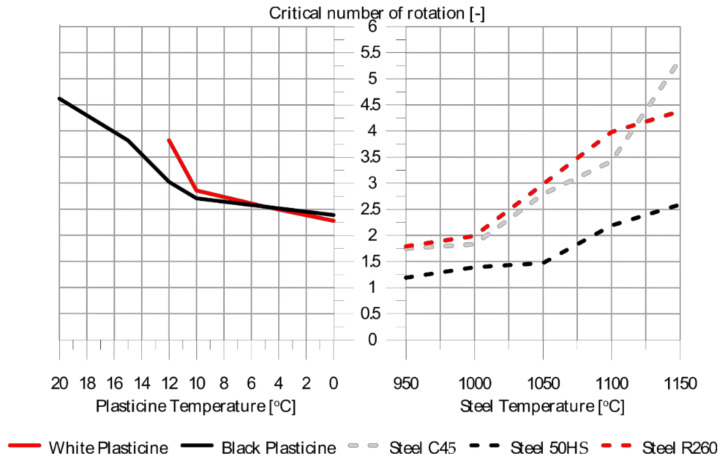
Comparative graph of changes in the limit rotation speed values for steel and model materials [58].

Comparison of the laboratory and real test results allowed us to determine the temperature ranges of the formed model materials, where they reflect the fracture process of the real materials. Figure 4 presents a graph showing the differences in the critical number of rotations at which cracking occurred inside the shaped sample.

Based on real and model tests, the possibility of using commercial white and black plasticine as model materials for the physical testing of hot rolling of steel was confirmed.

For model tests of comparative analysis of the two ball separation methods, commercial plasticine in two color variants (white and black) was used.

**Figure 5:**
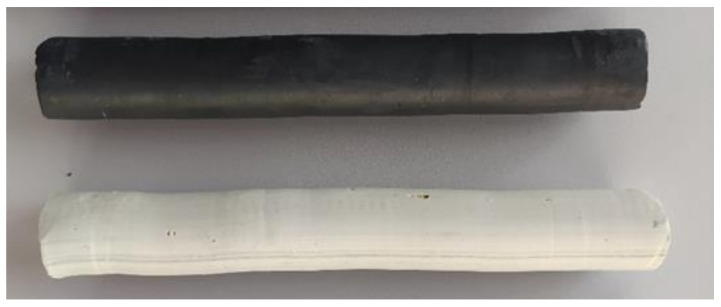
Samples used for model tests.

For each type of tool, three tests were performed. The workpiece was a rod with a diameter of 27.5 mm and a length of 250 mm. The workpieces are shown in Figure 5. Model tests were carried out at 0 °C with Teflon oil used as the lubricant.

## 3. Test Stand and Tools Used in Research

**Figure 6:**
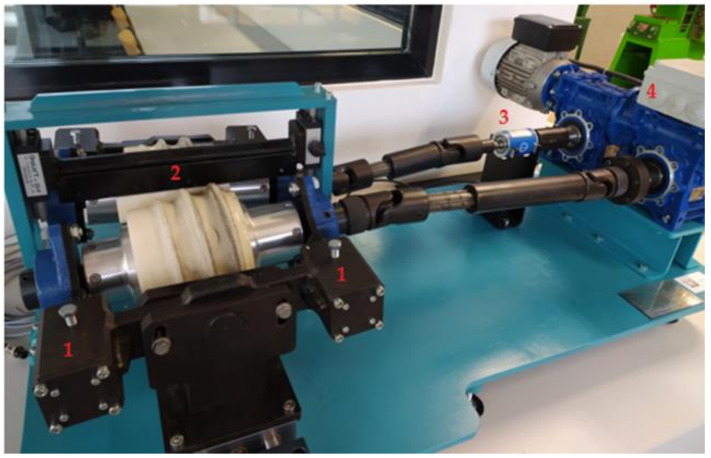
Model skew rolling mill, 1—expansion force sensors, 2—rolling cage, 3—torque sensor, 4—rolling mill drive unit.

The laboratory test stand for the physical modelling of skew rolling processes is constructed from three main components (Figure 6). The first one is the drive unit consisting of two worm reducers and an electric motor. The applied drive makes it possible to obtain the rotational speed of rolls equal to 15 revolutions per minute. The next mechanical unit is the transmission unit with an applied sensor for measuring the torque occurring on the rolling roll, which measures the torque during the plastic forming of the product. The largest component of the rolling stands is the rolling stand assembly. The rolling stand of the model rolling machine consists of two rollers, which have the possibility of changing the angle of twist between their axes in the range of −6 to 6°. In addition, two ways are used which have the ability to adjust the distance from the axis of rotation of the workpiece. The tools used in the model rolling mill are made from two parts printed on a 3D printer.

On one of the rolls, sensors measuring the force applied to the tools during the rolling process, were applied at locations marked with the number 1.

Two pairs of tools were used for the model tests. The first pair of tools has only a separating collar, which forms a knife to separate the balls, while the second set of tools used has two knives to cut off the bridge between the balls. The tools were set at a 4° angle to each other.

**Figure 7:**
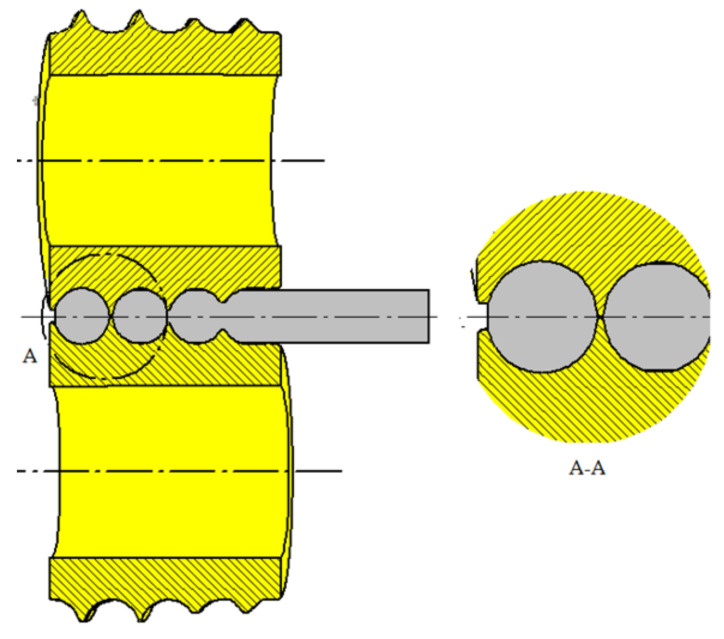
Ball splitting diagram on first type tools.

**Figure 8:**
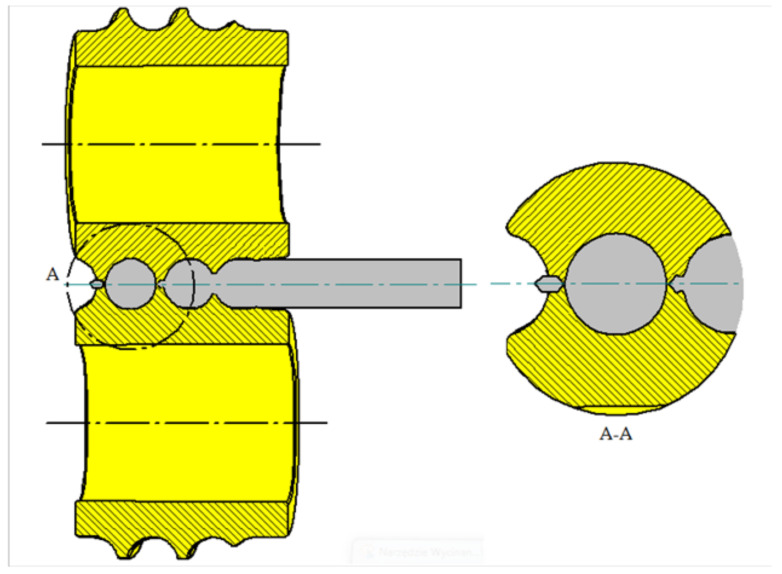
Ball splitting diagram on second type tools.

The first type of tool is shown in Figure 7, while in Figure 8, tools of the second type are shown.

In the case of type one tools, the workpiece material is rotated about its axis and gripped by the flange of the die. Next, the balls are separated by the expanding and approaching flange, which causes the material to flow from the bridge towards the finished balls, rolling it onto the ball surface.

In the case of type two tools, the workpiece is inserted between two rotating rollers. The expanding flange, as in the first set, shapes the charge and fills the tool blank with it. Type two tools differ in their method of ball separation. In this type of tool two knives are used, which have the function of separating the ball at the beginning from the bridge connecting it with the shaped charge, while the second knife at the end of the blank cuts off the bridge, which is a waste product. This creates a sphere with a smooth spherical surface.

**Figure 9:**
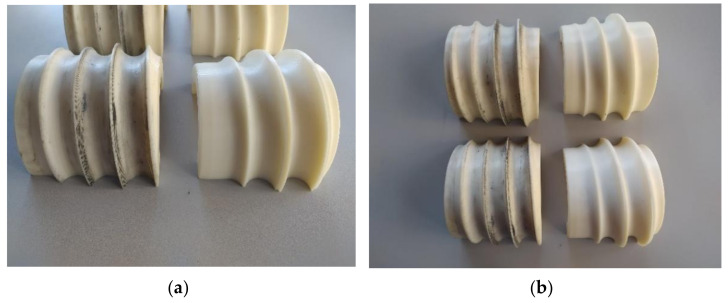
Tools used for model tests: (**a**) comparison of two different cutting methods; (**b**) top—a set of roller tools with one knife, bottom—a set of roller tools with two knives.

Figure 9 shows a summary of the tools used for the study. Figure 9a shows the differences between the two sets of tools. The tools used for skew ball rolling were made of ABS plastic. The geometry of the tools was made by 3D printing, which is based on the FDM printing system. FDM printing involves applying thin layers of molten material to form the geometry of the tool. The printing was done using a 3D uPrintSE machine from Stratasys (Rehovot, Israel).

ABS material is characterized by very high hardness and scratch resistance, but it is not resistant to contact with acids, alkalis and acetone.

P430 grade ABS material manufactured by Stratasys was used to print the tools. The characteristics of the material used are shown in Table 4.

## 4. Physical Modelling

**Figure 10:**
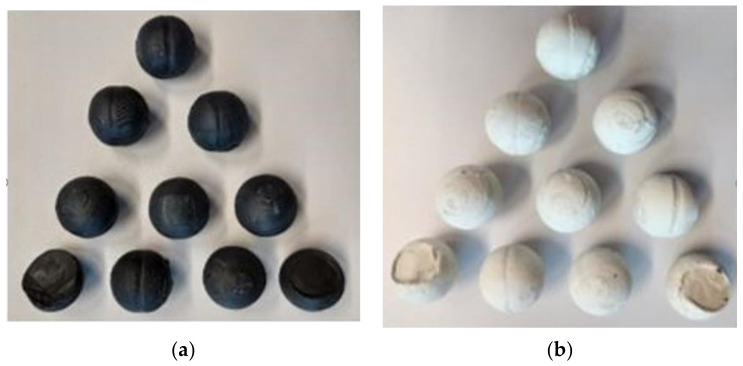
Samples made on type first tools: (**a**) black plasticine; (**b**) white plasticine.

**Figure 11:**
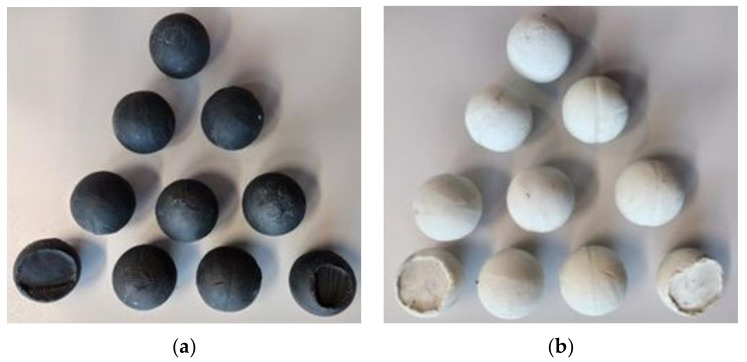
Samples made on type second tools: (**a**) black plasticine; (**b**) white plasticine.

Based on model tests of the skew rolling process, data were obtained, allowing for a comparative analysis of two types of tools with different ball separation systems. In the skew ball rolling process, eight full balls and two waste balls were obtained from each batch. The change in force and torque was recorded during the ball skew rolling process. Figure 10 and Figure 11 show photographs of the balls obtained.

Laboratory tests were carried out three times for each type of tool. The balls obtained were then dimensioned and the results obtained were collected in the form of a table. Table 5 and Table 6 show the results obtained from the measurement of one charge for each method and charge type.

The balls made on the first type tool set had the smallest diameter in the groove on the sphere circumference and it was approximately 28.16 ± 0.24 mm for white plasticine and approximately 28.06 ± 0.4 mm for black plasticine respectively, while the balls obtained by rolling with the second type tools had the groove on the sphere circumference with a very low profile. The smallest diameter value was for white plasticine balls approximately 28.25 ± 0.15 mm and for black plasticine balls it was approximately 28.26 ± 0.15 mm, respectively.

**Figure 12:**
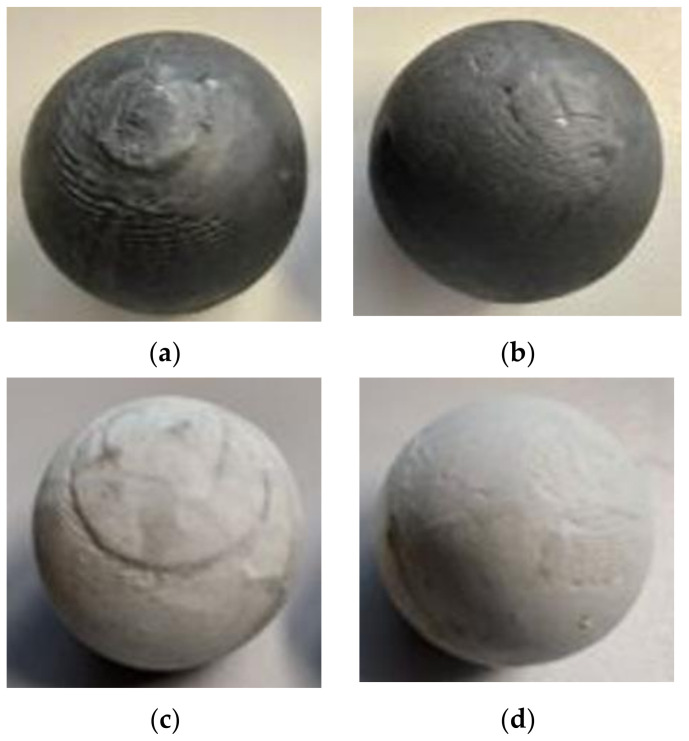
Comparison of balls obtained in the process of physical modelling: (**a**) I set of tolls black plasticine; (**b**) II set of tolls black plasticine; (**c**) I set of tolls white plasticine; (**d**) II set of tolls white plasticine.

The next step was a qualitative analysis of the surfaces of the spheres obtained. A comparison of spheres obtained by both methods is shown in Figure 12. Figure 12a,b shows a comparison of the surface quality of spheres obtained by the first and second methods from black model material, while Figure 12c,d shows a comparison of the surface quality of spheres from white plasticine.

**Figure 13:**
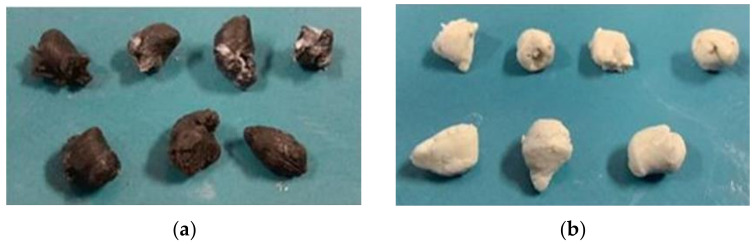
Cut-off bridges between balls during rolling: (**a**) black plasticine; (**b**) white plasticine.

Spheres obtained by rolling with a first type tool have a groove on the circumference of the sphere. Additionally, a build-up of material is formed at the poles of the balls obtained, which is the effect of the material being rolled remaining from the bridge connecting the balls during rolling. In contrast, balls obtained on type two tools have a smooth spherical surface with a very small or no groove around the circumference of the ball. The difference in the surface quality of balls rolled with type two tools is due to the use of two knives to cut off the bridge, which is treated as waste during rolling. The cut off bridges are shown in Figure 13.

**Figure 14:**
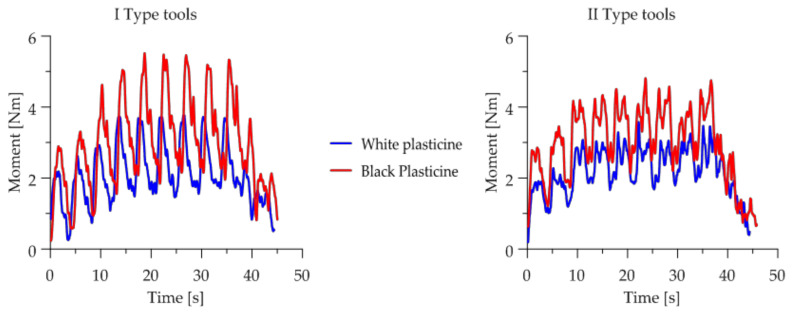
Tool roll torque change charts during rolling.

The next stage of the research was to analyze the change in torques during the skew ball rolling process. The torque curves are shown as diagrams in Figure 14.

The highest maximum torque, obtained with both types of tool, was observed during the shaping of black plasticine. The course of torque for the skew ball rolling process is a characteristic course for this process. The torque begins to increase until the process stabilizes, then the maximum torque values show a constant trend, while at the end of the process it begins to decrease again, such a course of change in torque values is due to the initial introduction of the charge. Then, the torque values stabilize when the blank is filled with material over the full length of the tool. A drop in torque values occurs when the last balls are removed from the tool.

It was also observed that the torque values during rolling on type one tools differ significantly from the rolling diagram of type two tools. In the case of type one tools, the torque increases rapidly during the rolling of a single ball, then drops to a certain level for the time remaining to roll it out.

The maximum torque obtained with the first type of tool was approximately 3.95 Nm for white plasticine and 5.8 Nm for black plasticine, while the values obtained with the second type of tool were lower: approximately 3.4 Nm for white plasticine and 4.5 Nm for black plasticine, respectively.

**Figure 15:**
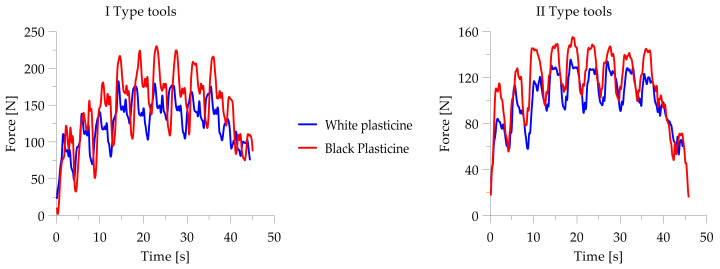
Diagrams of the change in the value of the expansion force during the ball skew rolling.

In the next part of the model tests, a qualitative and quantitative analysis of the change in the value of the strut force generated during skew ball rolling was carried out. Comparative plots of force values are shown in Figure 15. As in the case of the course of the change in the value of the torque, significant differences were observed between the process performed with tools of the first and second types. In the case of the use of the first set, the values of the forces are significantly higher than the forces obtained with the use of the second set. On the other hand, the course of the change of values as a function of the rolling time is similar to the courses of the torques.

The maximum force recorded during rolling with the first type of tools is on average equal to: white plasticine—184.9 N; black plasticine—approx. 230 N. On the other hand, the forces recorded during rolling with type two tools are about 135 N for white plasticine and about 150 N for black plasticine, respectively.

## 5. Real Test

The last stage of the research was to carry out a validation of the results obtained in the physical modelling method with the results obtained in the real process. For the analysis in the real process, tools of the first type with double the dimensions were used. In the real process, steel balls with a diameter of 57 mm were hot skew rolled. The feed material used in the rolling process was C60 steel. The steel used in the study is described by the constitutive equation represented by formula (3) [34]:

(3) $$\sigma_{F}=C_{1}e^{\left(C_{2}T\right)}\varepsilon^{\left(n_{1}T+n_{2}\right)}e^{\left(\frac{I_{1}T+I_{2}}{\varepsilon }\right)}{\dot{\varepsilon }}^{\left(m_{2}+m_{1}T\right)},$$

where σ_F_—flow stress [MPa]; ε—effective strain [-]; ε—strain rate [s^−1^]; *T*—temperature [°C]; *C_1_, C_2_, n_1_, n_2_, I_1_, I_2_, m_1_, m_2_*—constants presented in Table 7.

The real rolling stand was equipped with a torque sensor for one of the drive shafts.

The workpiece used for the real tests was a bar with a diameter of 55 mm and length of 500 mm. The rolling process was carried out for workpieces up to a temperature of 1050 °C. The workpiece bar was heated in an electric furnace.

**Figure 16:**
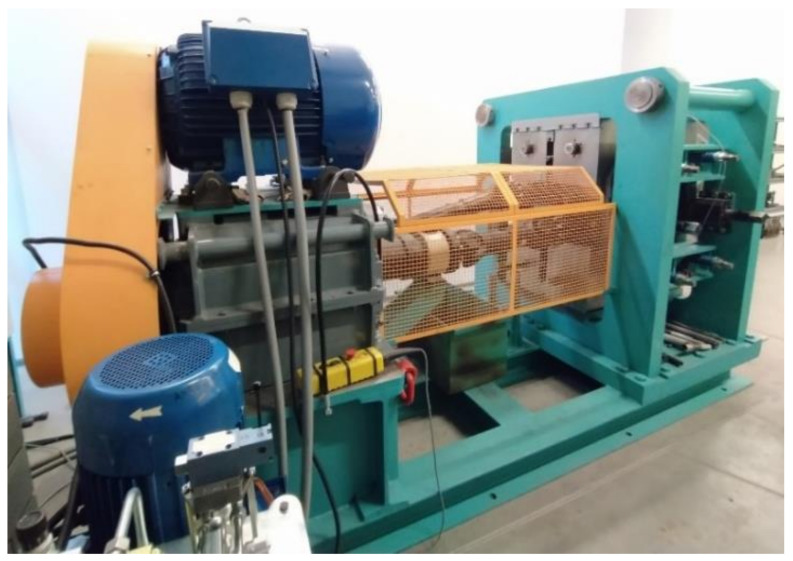
Laboratory skew rolling mill—the real machine.

**Figure 17:**
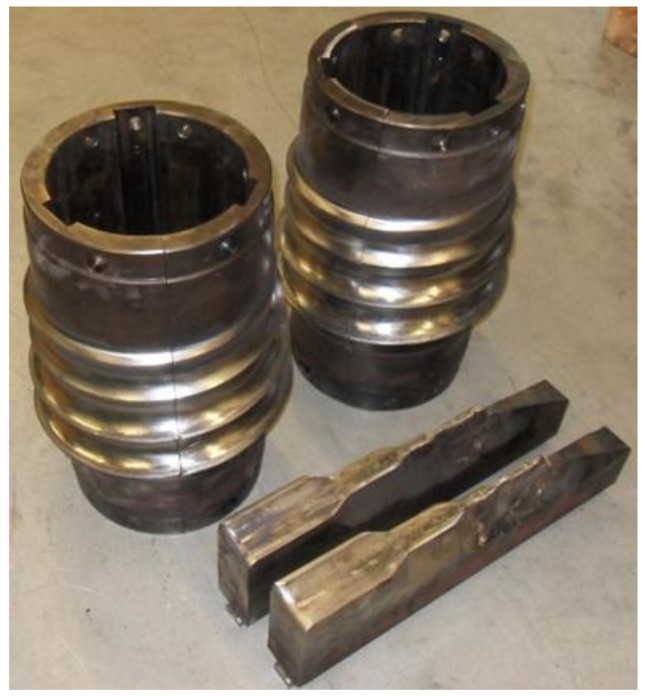
Second type tools for real research.

The helical roll tools used in the actual skew rolling mill (Figure 16) were set at an angle of 4° to each other (the same as for model rolling). The helical tools used for the actual tests are shown in Figure 17, each tool roll was divided into three segments.

Based on Equation (1), similarity coefficients were calculated for white and black plasticine formed at 0 °C to C60 steel processed at 1050 °C. The calculated coefficients are shown in Table 8.

Based on the results obtained from the skew rolling process and the real process, estimates of the torque values during rolling were calculated. Then, the estimated results obtained from the physical modelling were compared and contrasted with the results obtained from the real process. The calculations were carried out according to Equation (4) [34].

(4) $$M_{max}=\lambda M\prime_{max}s^{3}$$

where: *M’max*—maximum torque during model rolling, *λ*—similarity coefficient of the model material and the real material, *s*—scale of the real tool relative to the model one.

**Figure 18:**
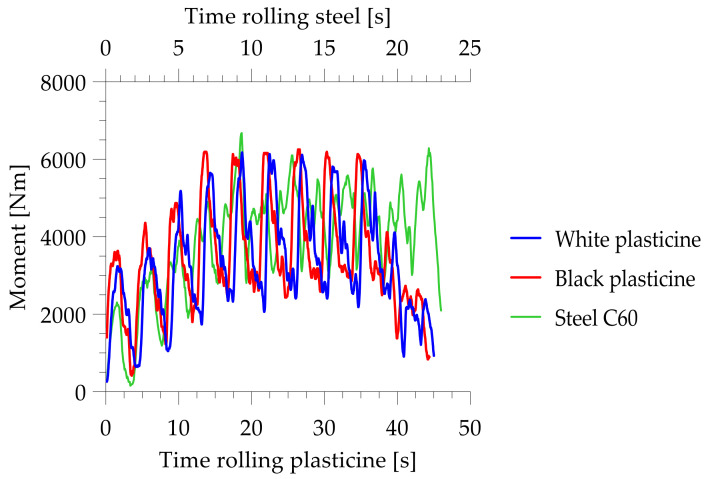
Diagram of torque variation during ball shaping.

Figure 18 shows a graph comparing the progression and variation of torque values during the ball rolling process. The graph shows the comparison of moments obtained during real rolling and model rolling.

As a result of the analysis of the data obtained in the model rolling process and the real ball rolling process, a very high agreement between the results of the model test and the real test was observed. The maximum moment obtained in the process of real hot rolling of steel balls at 1050 °C with a diameter of Ø57 mm was equal to 6650 Nm. The torques obtained by physical modelling show very good agreement with those obtained in the real process. Torques obtained by estimation based on the similarity of the flow curves showed that black plasticine modelled a torque of 6703Nm while white plasticine modelled a torque of 6950 Nm. Results with a smaller discrepancy value were obtained for black plasticine, for which the torque was approximately 0.7% higher, while for white plasticine the discrepancy was approximately 5%

On the basis of the obtained results of the torque estimates and their validation with the real process in which a high agreement of the results was observed, it was concluded that the carried out calculations of estimates of other parameters of the rolling process will also be characterized by a high coefficient of agreement. In addition, torque estimates for two-knife tools and calculations of expansion forces were carried out.

The calculation of the estimated expansion forces was carried out using relation (5) shown below [34].

(5) $$F_{max}=\lambda F\prime_{max}s^{2}$$

where: *F’max*—maximum value of force during model rolling, *λ*—similarity coefficient of model material and real material, *s*—scale of real tools against model tools.

Table 9 presents a summary of the estimated maximum values of moments and expansion forces calculated from the physical modelling.

**Figure 19:**
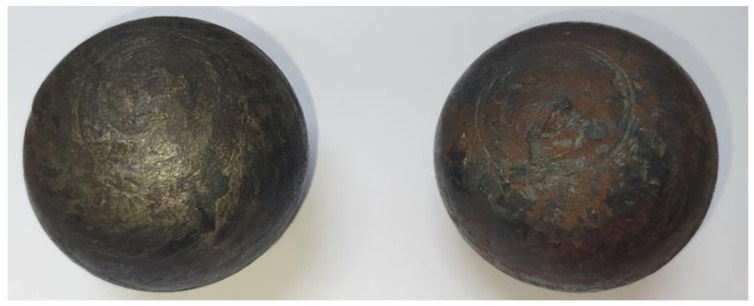
Steel balls Ø57 mm obtained by rolling.

After the test of the real process of hot skew rolling of balls, a comparative analysis of the surfaces of balls obtained by both test methods was carried out. The balls obtained from the real process are shown in Figure 19.

On the surface of the balls, the roughness of the spherical surface and a groove on the circumference of the balls were observed. In addition, material build-up residues were observed on the polar surfaces of the balls, which are the result of the material remaining from the bridge connecting the balls being rolled out. In the case of the surface quality of the balls obtained, a similarity was observed between the results of model tests using plasticine and the real process of hot rolling steel balls at 1050 °C.

## 6. Conclusions

The physical modelling studies carried out allowed comparison of the process of the skew rolling of balls with a diameter of 28.5 mm with two different sets of tools. The tools used differed in the way the balls were separated during rolling. Model tests of one of the analyzed cases were validated in the real process, which showed a very high similarity of the obtained results. As a result, it can be concluded that physical modelling allows for an accurate method of testing the skewed ball rolling process.

The analysis of model tests allowed us to define the following conclusions: the use of tools with double knives—type two tools resulted in an average torque reduction of 13.8%. In the case of rolling black plasticine, a torque reduction of about 15.2% was observed, while in the case of forming white model material, a torque reduction of 12.5% was observed in relation to forming these materials using tools with a single separating flange—type one tools.

The second type of tools reduces the maximum expanding force by an average of 29%. The use of tools with two knives reduced the maximum forces by 26% for white plasticine and 32% for black plasticine.

The use of tools of the second type makes it possible to obtain balls of much better shape and quality than those obtained by rolling with tools of the first type. Balls rolled with tools with a double knife are smoother because the formed bridge is cut off differently than in tools of the first type, where it is rolled out on the surface of the balls.

The use of the two-knife rolling method was found to be a better solution in terms of the magnitude of the forces, moments and ball surface area that are obtained.

The use of physical modelling in the comparative process of the two ball separation methods was found to be an accurate method.

On the other hand, the use of cutters has one drawback, which is that they wear out quickly, which would result in the need to use them in the form of replaceable segments.

In the case of ball rolling for bearings, where high product quality is required, tools with double knives can be used. However, in the case of balls used in grinding mills, where high quality is not required, tools of the first type can be used.

## Figures and Tables

**Table 1:** Constant of the white and black plasticine material models.

MATERIAL	C	N1	N2	M	B	A
White plasticine	0.48057	−0.0313	0.08705	0.2451	−0.0026	−0.03283
Black plasticine	0.6817	−0.0711	0.07203	0.2701	−0.0037	−0.07358

**Table 2:** Cockroft–Latham integral limits for PRIMO plasticine [56,57].

Temperature [°C]	Material	Cockcroft-Latham Integral Limits
0	White plasticine	0.646
	Black plasticine	0.691
5	White plasticine	0.786
	Black plasticine	1.34
10	White plasticine	1.27
	Black plasticine	1.25
15	White plasticine	1.38
	Black plasticine	2.04
20	White plasticine	1.45
	Black plasticine	2.00

**Table 3:** Friction coefficient values of the commercial plasticine—ABS tools node [58].

Temperature [°C]	White Plasticine			
	Dry		PTFE Oil	
	µ_s_	µ_d_	µ_s_	µ_d_
0	1.15	0.93	0.70	0.45
5	1.48	0.94	0.78	0.42
10	1.47	1.01	0.85	0.38
15	1.48	0.96	0.79	0.32
20	1.54	1.00	0.62	0.22
**Temperature** **[°C]**	**Black Plasticine**			
	**Dry**		**PTFE Oil**	
	**µ_s_**	**µ_d_**	**µ_s_**	**µ_d_**
0	1.32	0.97	0.90	0.44
5	1.40	0.96	0.92	0.37
10	1.46	0.97	0.87	0.36
15	1.67	1.01	0.93	0.38
20	1.75	1.06	0.52	0.20

**Table 4:** Material characteristics of the ABS P430 Stratasys.

Density [g/cm^3^]		1.04
Young’s module [GPa]		2.1
Thermal expansion coefficient [10−6/°C]		83.2
Maximum long working temperature [°C]		−40–80
Maximum short working temperature [°C]		100
Rockwell hardness		109.5
Mechanical Properties of the Print	Print along the height	Print along the horizontal edge
Bending strength [MPa]	58	35
Tensile strength [MPa]	32	27
Deformation to the yield point [%]	2	1
Deformation at crack [%]	6	3

**Table 5:** Dimensions of model balls obtained by skew rolling—I set of tools: D1—groove diameter, D2, D3—average ball in two planes.

White Plasticine				Black Plasticine			
D_1_	D_2_	D_3_	Avg.	D_1_	D_2_	D_3_	Avg.
mm	mm	mm	mm	mm	mm	mm	mm
I Set of Tools							
27.9	28.4	28.5	28.27	28.0	28.5	28.6	28,36
28.0	28.6	28.8	28.47	28.0	28.3	28.6	28.30
28.2	28.5	28.4	28.37	28.1	28.5	28.7	28.43
28.0	28.5	28.7	28.40	28.2	28.5	28.8	28.50
28.1	28.5	28.7	28.43	28.1	28.6	28.6	28.43
28.3	28.6	28.7	28.53	28.0	28.4	28.8	28.40
28.4	28.7	28.8	28.63	28.1	28.5	28.7	28.43
28.4	28.7	28.8	28.63	28.0	28.5	28.8	28.43
**28.16**	**28.56**	**28.68**	**Avg.**	**28.06**	**28.48**	**28.70**	**Avg.**

**Table 6:** Dimensions of model balls obtained by skew rolling—II set of tools: D1—groove diameter, D2, D3—average ball in two planes.

White Plasticine				Black Plasticine			
D_1_	D_2_	D_3_	Avg.	D_1_	D_2_	D_3_	Avg.
mm	mm	mm	mm	mm	mm	mm	mm
II Set of Tools							
28.2	28.2	28.7	*28.37*	28.3	28.4	28.8	*28.50*
28.3	28.3	28.5	*28.37*	28.2	28.3	28.6	*28.36*
28.2	28.4	28.9	*28.50*	28.2	28.2	28.5	*28.30*
28.2	28.3	28.6	*28.43*	28.2	28.3	28.7	*28.40*
28.4	28.4	28.8	*28.53*	28.2	28.2	28.4	*28.27*
28.2	28.3	28.5	*28.33*	28.3	28.3	28.5	*28.37*
28.1	28.2	28.8	*28.36*	28.4	28.4	28.6	*28.47*
28.4	28.4	28.7	*28.50*	28.3	28.3	28.7	*28.43*
**28.25**	**28.31**	**28.69**	**Avg.**	**28.26**	**28.30**	**28.60**	**Avg.**

**Table 7:** Constant of the C60 grade steel material models.

C1	C2	n1	n2	I1	I2	m1	m2
4857	−0.00374	−0.00022	0.07908	−0.000026	−0.0149	0.00016	−0.00965

**Table 8:** Similarity factors of the flow curves *λ* for the model material.

Materials	White Plasticine	Black Plasticine
Similarity factors *λ*	208	140

**Table 9:** Estimated values of rolling process parameters.

Parameters	I Set Tools		II Set Tools	
	White	Black	White	Black
Moment [Nm]	6.95	6.7	6.19	5.68
Force [kN]	153.8	129.7	113.98	87.9

## Data Availability

The data presented in this study are available on request from the corresponding author.
